# *ruvA* Mutants That Resolve Holliday Junctions but Do Not Reverse Replication Forks

**DOI:** 10.1371/journal.pgen.1000012

**Published:** 2008-03-07

**Authors:** Zeynep Baharoglu, Alison Sylvia Bradley, Marie Le Masson, Irina Tsaneva, Bénédicte Michel

**Affiliations:** 1 CNRS, Centre de Génétique Moléculaire, UPR 2167, Gif-sur-Yvette, France; 2 Université Paris-Sud, Orsay, France; 3 Université Pierre et Marie Curie-Paris 6, Paris, France; 4 UCL Department of Biochemistry and Molecular Biology, University College London, London, United Kingdom; Stanford UniversityUnited States of America

## Abstract

RuvAB and RuvABC complexes catalyze branch migration and resolution of Holliday junctions (HJs) respectively. In addition to their action in the last steps of homologous recombination, they process HJs made by replication fork reversal, a reaction which occurs at inactivated replication forks by the annealing of blocked leading and lagging strand ends. RuvAB was recently proposed to bind replication forks and directly catalyze their conversion into HJs. We report here the isolation and characterization of two separation-of-function *ruvA* mutants that resolve HJs, based on their capacity to promote conjugational recombination and recombinational repair of UV and mitomycin C lesions, but have lost the capacity to reverse forks. *In vivo* and *in vitro* evidence indicate that the *ruvA* mutations affect DNA binding and the stimulation of RuvB helicase activity. This work shows that RuvA's actions at forks and at HJs can be genetically separated, and that RuvA mutants compromised for fork reversal remain fully capable of homologous recombination.

## Author Summary

DNA replication is the process by which DNA strands are copied to ensure the transmission of the genetic material to daughter cells. Chromosome replication is not a continuous process but is subjected to accidental arrests, owing to the encounter of obstacles or to the dysfunctioning of a replication protein. In bacteria, inactivated replication forks restart but they are most often remodeled before restarting. Interestingly, enzymes involved in homologous recombination, the process that rearranges chromosomes, are also involved in fork-remodeling reactions. The subject of the present study is RuvAB, a highly conserved bacterial complex used as the model enzyme for resolution of recombination intermediates, which we found to also act at blocked forks. We describe here the isolation and characterization of *ruvA* mutants that have specifically lost the capability to act at inactivated replication forks, although they remain fully capable of homologous recombination. The existence of such *ruvA* mutants, their properties and those of the purified RuvA mutant proteins, indicate that the action of RuvAB at replication forks is more demanding that its action at recombination intermediates, but have nevertheless been preserved during evolution.

## Introduction

**Figure 1:**
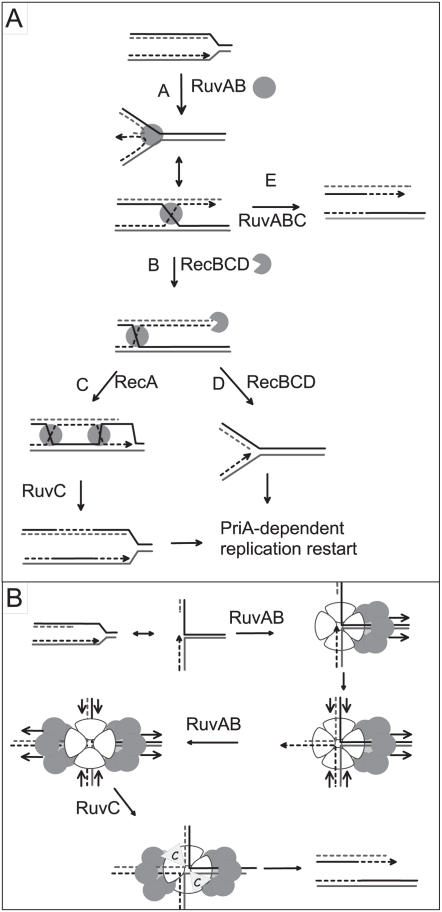
Model for replication fork reversal in a *dnaEts* mutant (adapted from [8],[20]). In the first step (A), the replication fork is arrested by inactivation of *dnaE*. RuvAB catalyzes the annealing of leading and lagging strand ends, *i.e.* fork reversal. The reversed fork forms a four-arm structure (Holliday junction, HJ; two alternative representations of this structure are shown, open X and parallel stacked X). RecBC is essential for resetting of the fork, either by RecA-dependent homologous recombination (B–C) or by DNA degradation (B–D). In the absence of RecBCD (E), resolution of the HJ causes chromosome linearization. Continuous lines: parental chromosome. Dashed lines: newly-synthesized strands. Circle: RuvAB. Incised circle: RecBCD. B: Model of RuvAB action at blocked forks. In the first step, a RuvA tetramer binds to the fork and drives the assembly of a RuvB hexamer on the template strands. The translocase action of this RuvB hexamer pulls the leading and lagging strands into the RuvA complex (direction of migration of DNA is indicated by arrows) and results in the formation of a HJ. This HJ is bound by a second RuvB hexamer forming a bona fide branch migration complex (direction of translocation of DNA is indicated by arrows, it is unclear at present whether the active form of the branch migration complex in vivo carries one or, as drawn here, two tetramers of RuvA). HJ resolution by RuvC results in a cleaved replication fork.

DNA replication and recombination are two processes that are now recognized as more closely connected than originally suspected. It is well documented that replication defects induce the formation of recombination substrates, such as double-stranded DNA ends or single-stranded DNA regions (ssDNA). Depending on the nature of the replication defect, such recombinogenic DNA structures form at blocked replication forks and/or behind forks, on the newly replicated daughter chromatids ([1]; reviewed in [2]–[4]) (Figure 1A). In addition, replication and recombination can be directly coupled by enzymes that recognize two different targets, one specifically produced during replication and the other during recombination. The best-documented example is the bacterial PriA protein, which promotes replication restart (i) independently of recombination by its virtue of recognizing replication forks and (ii) during double-stranded DNA end recombinational repair by its virtue of recognizing D-loop structures (reviewed in [5]). Another example is the RuvAB complex, originally identified for its activity on Holliday junctions (HJs), four-DNA arm recombination intermediates (reviewed in [6],[7]), and recently proposed to also act on inactivated replication forks [8] (Figure 1).

RuvA and RuvB are nearly ubiquitous bacterial proteins, with a well-conserved structure and function in distantly related species [9]–[11]. During homologous recombination, a RuvA tetramer binds a HJ formed by RecA-catalyzed strand exchange and two RuvB hexamers assemble on two opposite arms of the HJ to form the tripartite RuvAB-HJ complex. RuvB belongs to the AAA+ (ATPase Associated with various cellular Activities) family of enzymes and acts as a molecular motor for branch migration. Binding of the dimeric endonuclease RuvC leads to the formation of a RuvABC complex that resolves HJs to produce recombinant molecules. Band shift experiments and structural studies of RuvA complexes with synthetic HJs indicate that two tetramers can eventually assemble to sandwich a HJ and form an octameric RuvA complex [12]–[15]. RuvABC are essential for recombinational repair of DNA lesions in bacteria, and the *ruvA ruvB* operon is induced by DNA damage, *via* the SOS response [16].

In addition to its crucial role in processing HJs during homologous recombination, RuvAB binds fork structures *in vitro* [17]–[19], and was recently proposed to act at certain inactivated replication forks *in vivo* [8]. Indeed, inactivated replication forks that occur in several replication mutants are converted into HJs by the annealing of newly synthesized leading and lagging strand ends, a reaction called replication fork reversal (RFR) [20]; reviewed in [3],[4] (Figure 1A). HJs formed by RFR, as those formed by homologous recombination, are resolved by RuvABC. Notably, RuvAB was shown to be essential for the formation of HJs at blocked forks in some replication mutants, including the *dnaEts* mutant affected for the catalytic subunit of the main *E. coli* DNA polymerase Pol III. We proposed that RuvAB binds to certain inactivated replication forks and catalyzes their conversion into HJs [8] (Figure 1B).

As the two functions of RuvAB in *E. coli*, resolution of HJs and RFR, involve interactions with two different target molecules, we searched for mutants that have lost only one of these functions. We describe here the isolation and characterization of two *ruvA* mutants that still promote homologous recombination while they have lost the capacity to reverse forks.

## Results

### Selection of UV^R^ ruvA Mutants Defective in RFR

The *ruvA100*::Cm^R^ mutant is sensitive to UV irradiation, and UV resistance is restored in the presence of pGB-RuvA^+^ while the pGB2 vector has no effect (Table 1). The UV sensitivity of the *ruvA*, *ruvB* and *ruvC* mutants results from the lack of resolution of recombination intermediates, therefore reflects the recombination defect of these mutants [2],[21]. The products of a *ruvA* mutagenic PCR were cloned in pGB2, used to transform the *ruvA100* mutant, and the UV sensitivity of cells carrying recombinant plasmids was monitored. Seventeen clones were tested, nine remained UV sensitive, therefore contained a plasmid unable to complement the UV repair defect of the *ruvA* mutant. The remaining eight recombinant plasmids that carry a *ruvA* allele functional for UV repair were isolated and tested for their capacity to promote RFR in a *dnaEts* mutant (*dnaE486ts*, Table S1).

**Table 1:** *ruvA* mutant alleles deficient for RFR.

Plasmid	UV^a^	cfu^b^	% linear DNA^c^	Mutations
pGB2	0.00005	+	10.3±1.6 (5)	
pGB-RuvA+	0.9	−	60.7±1.2 (8)	
pGB-RuvAz26	0.2	+	38.4±1.5 (3)	E68G H136R
pGB-RuvAz60	0.7	+	16±2.7 (10)	H29R E40G Q58R K129E F140S S177G D184N
pGB-RuvAz80	0.2	+	12.1±1.1 (4)	H29R E40G E68G K129E F140S S177G D184N
pGB-RuvAz87	0.3	+	32.6±1.2 (3)	N79D N100D

a Survival of *ruvA100* mutant (JJC2971) containing the different plasmids after UV irradiation at a dose of 40 Joules/m2.

b Colony forming units at 37°C of *dnaEts recF recB ruvA100* (JJC3110) containing the different plasmids.

c Percentage of DNA entering pulse field gels in *dnaEts recF recB ruvA100* cells (JJC3110) containing the different plasmids (42°C). (N) indicates the number of independent experiments.

The *dnaE486ts* mutant is completely defective at 42°C, and partially affected at 37°C for the main *E. coli* DNA polymerase, Pol III. Because its slight growth defect at 37°C is suppressed by preventing SOS induction [22], *recF* derivatives of *dnaEts* were used for the screening of RFR deficient *ruvA* mutants. RFR takes place at *dnaEts*-blocked forks and renders RecB essential for viability. Consequently, growth of a *dnaEts recF* mutant at 37°C is prevented by *recB* inactivation [23]. However, because RFR requires RuvAB, inactivation of *ruvA* or *ruvB* restores the growth of *dnaEts recB recF* mutants and introduction of a functional *ruvA* gene in a *dnaEts recF recB ruvA100* mutant is lethal [8] (Table 1). Three of the plasmids conferring UV^R^ to a *ruvA100* mutant were lethal in *dnaEts recF recB ruvA100* cells at 37°C, therefore presumably carried a wild-type *ruvA* gene. The other five plasmids allowed variable levels of viability and therefore expressed candidate RFR-defective RuvAs. To ascertain whether the *ruvA* alleles in these plasmids were deficient for RFR, fork breakage was measured directly.

In *dnaEts recB* mutants, resolution of HJs formed by RFR leads to an increase in the level of linear DNA *in vivo*, which can be quantified by pulse field gel electrophoresis (PFGE), as only linear DNA can enter PFG [20]. Because RuvAB promotes fork reversal and RuvABC resolves the resulting HJ, the level of linear DNA resulting from fork breakage is high in *dnaEts recF recB* cells (∼60%) and low in the *dnaEts recF recB ruvA100* mutant (∼10%) [8] (Table 1). As expected, fork breakage was increased in *dnaEts recF recB ruvA100* cells by the presence of pGB-RuvA^+^ (Table 1). Fork breakage remained low in the presence of 4 candidate plasmids: 12–16% for pGB-*ruvAz60* and pGB-*ruvAz80*, 32–38% for pGB-*ruvAz26* and pGB-*ruvAz87* (Table 1). These *ruvA* alleles can promote HJ resolution, since they fully complement the UV sensitivity of a *ruvA* null mutant. Therefore, the defect in fork breakage suggests that they are affected for fork reversal.

**Figure 2:**
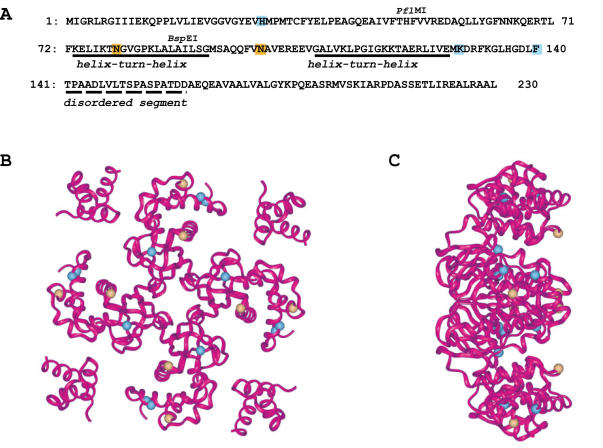
Positions of *ruvAz3* and *ruvA87* mutations. The mutations in RuvAz3 and RuvAz87 on the primary sequence (A) are shown in blue (H29R K129E F140S) and in yellow (N79D N100D), respectively. Full and dashed lines indicate the positions of the two helix-turn-helix in domains II and of the disordered segment that separates domains II and III, respectively [33]The positions of the three restriction sites used to separate the mutations in the original *ruvAz60* allele are shown above the sequence. Domain I (1 to 64), II (65–140) and III (156–203) are not indicated. The mutations are also shown as blue (RuvAz3) or yellow (RuvAz87) spheres in a ribbon view of the 3D structure of RuvA, viewed at the DNA-binding face (B) and a perpendicular side view of this (C).

Sequencing of *ruvA* on pGB-*ruvAz26* and pGB-*ruvAz87* showed that these were double mutants (E68G H136R and N79D N100D, respectively). pGB-*ruvAz60* and pGB-*ruvAz80* carried 7 mutations each, 6 of which were identical (Table 1). This result was not surprising since all plasmids derived from the same PCR cloning experiment. In order to identify the mutations in *ruvAz60* which are necessary and sufficient to abolish RFR, the 7 mutations were introduced individually or in combination on a pGB-*ruvA* plasmid (see Supplementary Material). The capacity of the *ruvA* mutant alleles to promote homologous recombination was monitored by measuring the UV resistance that they confer to *ruvA100* cells. Their inability to catalyze RFR was deduced from the viability and the low level of fork breakage that they confer to *dnaEts recF recB ruvA100* mutants (Table 2). Sub-cloning of different *ruvA* gene regions and site-directed mutagenesis showed that three mutations were necessary and sufficient for the RFR defect (H29R K129E F140S, pGB-*ruvAz3*, Table 2; Figure 2). pGB-*ruvAz3* and pGB-*ruvAz87* (N79D N100D) were used for further studies.

**Table 2:** Three mutations in pGB-*ruvAz60* are necessary and sufficient to inactivate RFR.

Plasmid	Mutations	UV^a^	cfu^b^	% linear DNA^c^
pGB2		0.00002	+	10.3
pGB-RuvA+		0.9	−	58.2±2.2
pGB-*ruvAz60*	H29R E40G Q58R K129E F140S S177G D184N	0.2 to 0.5	+	14.1±0.5
pF1+	Q58R K129E F140S S177G D184N	0.2 to 0.5	variable	22.6±4
pF2+	H29R E40G K129E F140S S177G D184N	0.2 to 0.5	+	17.6±0.9
pF3+	H29R E40G Q58R S177G D184N	0.2 to 0.5	−	50.4±1
pF4+	H29R E40G Q58R K129E F140S	0.2 to 0.5	+	14.7±0.6
pF1m	H29R E40G	0.2 to 0.5	−	48±3.2
pF2m	Q58R	0.2 to 0.5	−	65.2±6.9
pF3m	K129E F140S	0.2 to 0.5	−	47.2±2
pF4m	S177G D184N	0.2 to 0.5	−	56.5
pF1m-F3m	H29R E40G K129E F140S	0.2 to 0.5	+	23.6±3
pGB-*ruvAz3* d	H29R K129E F140S	0.2 to 0.5	+	15.5±1.7
pF1m1-F3m1	H29R K129E	0.2 to 0.5	−	39.6±10
pF1m1-F3m2	H29R F140S	0.2 to 0.5	+/−	45.8±3.6
pF1m1-F3m1 mut11^e^	E11G H29R K129E	0.2 to 0.5	+	22.4±1.4
pF1m2-F3m	E40G K129E F140S	0.2 to 0.5	+/−	33.2±0.1

a Survival of *ruvA100* mutant (JJC2971) containing different plasmids after 40 Joules/m^2^ UV irradiation.

b Plating of *dnaEts recF recB ruvA100* (JJC3110) containing different plasmids at 37°C. (+) 50 to 100% plating efficiency, (−) less than 0.01% plating efficiency, (+/−) or (+/−) 0.01 to 10% plating efficiency.

c Percentage of DNA entering pulse field gels in *dnaEts recF recB ruvA100* cells containing different plasmids (42°C). Results are the average of 2 or 3 independent experiments except pGB-*ruvAz3* (H29R K129E F140S) which was tested 5 times, pF1m-F3m (H29R E40G K129E F140S) 7 times, and pF4m (S177G D184N) which was tested once.

d This triple mutant was called *ruvAz3* and was used for further analysis.

e This mutant was fortuitously obtained during the construction of pF1m1–F3m1 by site directed mutagenesis.

A *recF* mutant background was used for the original screening experiment because inactivation of *recF* improves the viability of *dnaEts recB ruvA* cells and we have shown that the *recF* mutation has no effect on RFR in *dnaEts* cells [8]. As expected, the *ruvA* alleles identified as deficient for RFR in a *recF* null background were also unable to promote fork breakage in the presence of RecF (*dnaEts recB ruvA100*, pGB-*ruvAz3* and pGB-*ruvAz87*, Table 3). A RecF^+^ context was therefore used for the subsequent experiments.

**Table 3:** Increasing the amount of RuvB restores RFR in *ruvAz* mutants.

Strain	Relevant genotype	Plasmid	% linear DNA
JJC3723	*dnaEts recB ruvA100*	none	4.8±0.9 (3)
JJC3723	*dnaEts recB ruvA100*	pGB2	11.2±0.3 (2)
JJC3723	*dnaEts recB ruvA100*	pGB-RuvA^+^	55±3 (3)
JJC3723	*dnaEts recB ruvA100*	pGB-*ruvAz60*	6.1±0.35 (2)
JJC3723	*dnaEts recB ruvA100*	pGB-*ruvAz3*	6.5±0.7 (4)
JJC3723	*dnaEts recB ruvA100*	pGB-*ruvAz87*	14±1.3
JJC3723	*dnaEts recB ruvA100*	pGB-*ruvAz3*-RuvB^+^	63.4±1.1 (3)
JJC3723	*dnaEts recB ruvA100*	pGB-*ruvAz87*-RuvB^+^	69±4.9 (3)
JJC3939 JJC4015 JJC4016	*dnaEts recB ruvAz60*	None	60.5±5.8 (4)
JJC4196	*dnaEts recB ruvA100 rus-1*	pGB2	33.1±4.6 (3)
JJC4196	*dnaEts recB ruvA100 rus-1*	pGB-RuvA^+^	54.3±2.1 (2)
JJC4196	*dnaEts recB ruvA100 rus-1*	pGB-*ruvAz3*	40.5±7 (6)
JJC4196	*dnaEts recB ruvA100 rus-1*	pGB-*ruvAz87*	27.3±2.9 (5)
JJC4196	*dnaEts recB ruvA100 rus-1*	pGB-RuvA^+^-RuvB^+^	70.6±7.1 (2)
JJC4196	*dnaEts recB ruvA100 rus-1*	pGB-*ruvAz3*-RuvB^+^	67.3±2.6 (3)
JJC4196	*dnaEts recB ruvA100 rus-1*	pGB-*ruvAz87*-RuvB^+^	70.3±2.1 (3)

(N) indicates the number of independent experiments.

### The ruvA Mutations Prevent RFR Only When ruvA is Expressed in Limiting Amounts

Although *ruvA* and *ruvB* genes form an operon, *ruvB* is expressed in the *ruvA100*::Cm^R^ mutant, as only wild-type RuvA protein is required for suppression of the recombination defects (Table 1). In *dnaEts recB ruvA100* [pGB-*ruvA*] mutants, *ruvB* is expressed from the chromosomal locus downstream of the *ruvA100*::Cm^R^ insertion, whereas *ruvA* is expressed from its own promoter on the plasmid which has about 10 copies per cell. The imbalance between *ruvA* and *ruvB* expression could play a role in the RFR defect conferred by *ruvAz3* and *ruvAz87* mutations. To test this possibility, we cloned *ruvB* downstream of these *ruvA* alleles on the pGB-*ruvAz3* and pGB-*ruvAz87* plasmids. Co-expression of *ruvB* restored a high level of breakage in *dnaEts recB ruvA100* cells expressing *ruvAz3* or *ruvAz87* (Table 3). In addition, a high level of breakage was observed when the *ruvAz60* allele was inserted into the chromosome (Table 3). These observations indicate that the mutant RuvAz-RuvB complexes are defective for RFR only if the *ruvB* gene is expressed from a single chromosomal copy downstream of the *ruvA100*::Cm^R^ mutation. The insertion of the Cm^R^ gene in *ruvA* most likely reduces the amount of RuvB protein synthesized.

### ruvAz3 and ruvAz87 Mutants Catalyze Homologous Recombination in Various Contexts

**Figure 3:**
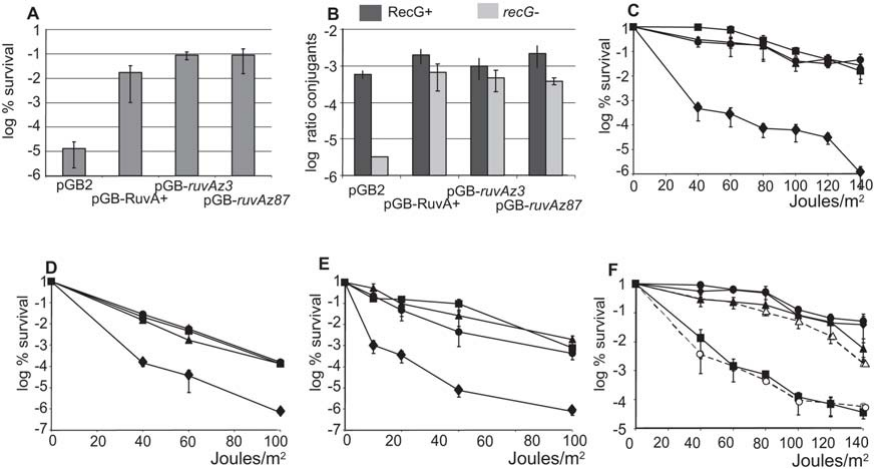
ruvAz3 and *ruvA87* suppress the recombination defect of a *ruvA100* mutant. (A) Exponentially growing JJC 2971 (*ruvA100*) cells containing different plasmids were treated with 2 µg/ml mitomycin C for 90 min, plated on LB-spectinomycin and incubated over-night. Ratios of colony forming units (cfu) in treated *vs* untreated cultures are shown. (B) Exponentially growing cells were mixed with a His^+^ Hfr donor for 25 min, plated on chloramphenicol minimal medium devoid of histidine and incubated for 48 hours. Ratios of His^+^ *vs* total recipient cfu are shown. Recipient RecG^+^ JJC2971 (*ruvA100*), recipient *recG* ^−^ JJC3207 (*ruvA100 recG::kanR*). (C) Appropriate dilutions of exponentially growing JJC 2971 (*ruvA100*) cells containing different plasmids were plated on LB-spectinomycin, UV-irradiated, and incubated over-night. Ratios of cfu on irradiated *vs* non-irradiated plates were calculated. Average of at least three values and standard deviations are shown. Diamonds: pGB2, squares: pGB-RuvA^+^, circles: pGB-ruvAz3, triangles: pGB-ruvAz87. (D) Same experiments with JJC3375 (*ruvA100 recR*), symbols are as in panel C. (E) Same experiments with JJC3207 (*ruvA100 recG*), symbols are as in panel C. (F) same experiments with JJC2761 (Δ*ruvABC rus-1*), closed symbols are as in panel C, dashed line-open circles: pGB-ruvAz3-RuvB^+^, dashed line-open triangles: pGB-ruvAz87-RuvB^+^.

The capacity of *ruvAz* alleles to catalyze homologous recombination was analyzed by different assays. Mitomycin C is a DNA damaging agent that causes various DNA lesions and mitomycin C treatment prevents growth of a *ruvA* null mutant, defective for recombinational repair [24] (Figure 3A). Introduction of pGB-*ruvAz3* or -*ruvAz87* plasmids in the *ruvA100* mutant restored the same level of resistance to mitomycin C treatment as pGB-RuvA^+^ (Figure 3A), indicating that these mutant *ruvA* alleles promote recombinational repair of mitomycin C lesions. Inactivation of *ruvA* decreased conjugational recombination about 3–5 fold [25] (Figure 3B); this defect was also suppressed by the *ruvAz3* or *ruvAz87* alleles (Figure 3B, RecG^+^).

Full suppression of the UV sensitivity of single *ruvA100* mutants by pGB-*ruvAz3* and -*ruvAz87* plasmids was observed at a wide range of UV doses (Figure 3C), and at 42°C, the temperature used for fork-breakage measurements (data not shown). The UV resistance conferred by these alleles was also tested in different mutant backgrounds. The *ruvA100* mutation decreases the survival of UV-irradiated *recR* mutants deficient for the recombinational repair of gaps (Figure 3D). Introducing pGB-*ruvAz3* or -*ruvAz87* plasmids fully suppressed the UV-repair defect caused by the *ruvA100* mutation in a *ruvA100 recR* double mutant (Figure 3D). *recG* inactivation affects the viability of *ruvA*, *ruvB* or *ruvC* mutants and renders them extremely deficient for homologous recombination [25] (Figure 3F). It was proposed that RecG provides an alternative way of resolving HJs *in vivo* [26]. Expression of *ruvAz3* or *ruvAz87* in a *ruvA100 recG* double mutant suppressed the viability defect (not shown) and the sensitivity to UV irradiation (Figure 3E). Accordingly, conjugational recombination was not significantly different in *ruvA100 recG* mutants carrying pGB-RuvA^+^, -*ruvAz3* or -*ruvAz87* (Figure 3B, *recG^−^*). These findings indicate that the mutant Ruv proteins promote HJ resolution in a *recG* context.

### ruvAz3 and ruvAz87 Mutants are Affected for HJ Binding in Vivo

RusA is a HJ resolvase that is only expressed in *E. coli* when the *rusA* ORF is activated by the insertion of an upstream IS element (*rus-1* mutant, [27]). RusA resolves HJs *in vitro* but is devoid of detectable branch migration activity [28]. By allowing RusA resolvase synthesis, the *rus-1* mutation suppresses the recombination defects of *ruvA* mutants *in vivo*. However, suppression is partial in *ruvC* mutants in which RuvA protects HJs from RusA action [27]. As shown in Figure 3F, Δ*ruvABC rus-1* cells were resistant to UV irradiation as expected (HJs are resolved by RusA), and expression of wild-type RuvA from pGB2-RuvA^+^ made them UV sensitive (RusA-catalyzed resolution is prevented by RuvA binding to HJs). In contrast with pGB-RuvA^+^, plasmids carrying the *ruvAz3* or *ruvAz87* allele did not compromise the survival of *ruvA100 rus-1* UV-irradiated cells, suggesting that these mutant RuvA proteins are not capable of protecting recombination intermediates from resolution by RusA (Figure 3F). Co-expression of *ruvB* and *ruvAz87* only slightly prevented RusA action, suggesting that the HJ-binding defect of the RuvAz87 protein is mainly independent of the amount of RuvB. In contrast, co-expression of *ruvB* and *ruvAz3* fully prevented RusA action (Figure 3F). This observation indicates that the HJ-binding defect of RuvAz3 can be suppressed by increasing the cellular level of RuvB, suggesting a defect in RuvAz3-RuvB protein interactions.

Expression of RusA allows HJ resolution in *dnaEts recB ruvA100 rus-1*, but, because RuvA is required for RFR, the level of linear DNA remains significantly lower in the absence of RuvA than in its presence (compare JJC4196 containing pGB2 and pGB-RuvA^+^, Table 3; Baharoglu et al, 2006). RuvAz3 and RuvAz87 did not restore a high level of linear DNA in the presence of RusA, unless these *ruvAz* alleles were made capable of RFR by the presence of *ruvB* on the plasmid (compare JJC4196 containing pGB2-*ruvAz* and pGB-RuvA^+^, Table 3). This observation confirms that the fork breakage defect in the presence of RuvAz3 or RuvAz87 does not result from a defect in HJ resolution, but rather from a defect in HJ formation. A slight increase in the percentage of DNA entering PFG is observed upon RusA expression (compare JJC3723 and JJC4196 containing pGB2, Table 3). This may result, at least in part, from RusA-resolution of HJs made behind replication forks by recombination at gaps, which would preventing linear DNA migration if left unresolved [8],[29].

### In Vitro Properties of Purified RuvA-z3 and RuvAz87 Proteins

**Figure 4:**
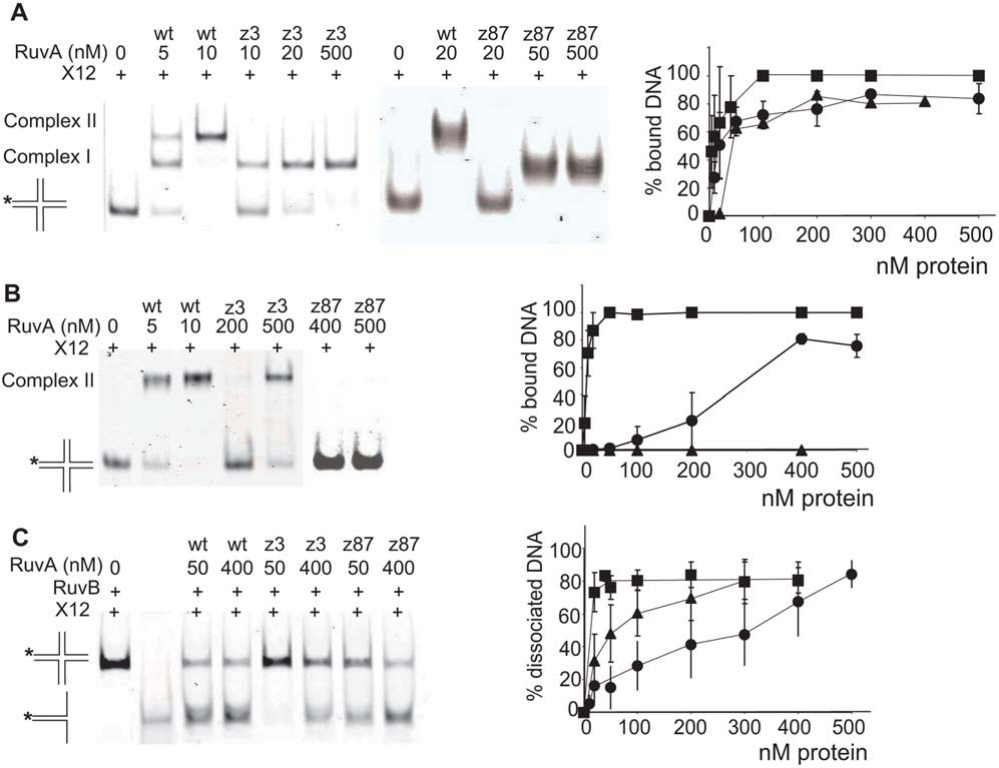
RuvAz3 and RuvAz87 proteins are deficient for octamerisation on HJs and slightly affected for HJ branch migration. (A) binding assay: Fluorescence-labeled junction ×12 (∼4 ng) was incubated in the presence of 5 mM EDTA with varying amounts of wild-type (wt) RuvA, RuvAz3 or RuvAz87, as indicated. Binding curves were obtained by quantification using Li-cor Biosciences ODYSSEY infrared imaging system. Squares: RuvA, circles: RuvAz3, triangles: RuvAz87. (B) as in A but in the presence of 3 mM Mg^2+^ (in the absence of EDTA). (C) branch migration assay: Reaction mixtures containing ∼4 ng labeled synthetic ×12 junctions were incubated withf 250 nM RuvB and various amounts of wild-type or mutant RuvA, as indicated. Lane 2 is the substrate only. Gels were quantified as in A. Symbols are as in A.

Wild-type RuvA and mutant RuvAz3 and RuvAz87 proteins were over-expressed in *E. coli* and purified. Both mutants behaved as wild type at all chromatographic steps during purification. The oligomeric states of the mutant RuvA proteins were analyzed by SDS-PAGE without boiling the protein samples [30]. Both RuvAz3 and RuvAz87 showed a tetramer band under denaturing gel conditions (data not shown), which indicates that the structural organization of the RuvA mutants was not affected by the mutations. Binding to a substrate that mimics a Holliday junction (X12) was measured by electrophoretic mobility shift assays (EMSA). In the presence of EDTA, wild-type RuvA formed two complexes: complex I, which contains one bound RuvA tetramer per HJ and complex II, in which the junction is sandwiched between two tetramers [31],[32] (Figure 4A). The proportion of DNA bound by RuvAz3 and RuvAz87 proteins was only slightly lower than that with the control wild-type RuvA protein. However, both mutant RuvA proteins could only form complex I (Figure 4A). In the presence of Mg^2+^, where only octameric complexes can be observed [31], RuvAz3 was partially and RuvAz87 totally unable to promote band-shifts (Figure 4B). Therefore, both proteins are slightly affected for binding to HJ DNA, and strongly affected for the formation and/or the stability of octamers on the junction.

The mutant RuvA proteins were tested for branch migration of ×12 in the presence of wild-type RuvB protein (Figure 4C). Both mutants were able to support branch migration of ×12 but at significantly higher concentrations than wild type RuvA. RuvAz87 exhibited a relatively high branch migration activity with RuvB, while RuvAz3 was more defective in branch migration. Therefore RuvB compensates for the binding defect of RuvAz87 but only partially for that of RuvAz3. These results indicate that whereas RuvAz87 is more affected than RuvAz3 for HJ binding, RuvAz3 is more affected for RuvB binding and/or activation.

**Figure 5:**
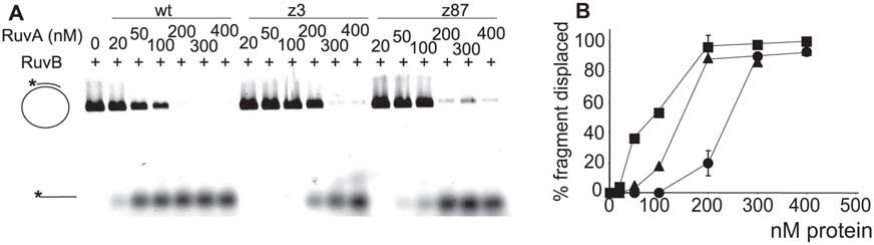
RuvAz3 is more deficient for the stimulation of RuvB helicase activity than RuvAz87. DNA helicase substrate consisting of fluorescence-labeled 52mer oligonucleotide annealed to ϕX174 single-stranded DNA (4 ng) was incubated with 250 nM RuvB and various amounts of wild-type or mutant RuvA, as indicated. Gels were quantified as in Figure 4. Symbols are as in Figure 4.

To confirm this idea, RuvB helicase activity was compared in the presence of wild-type and mutant RuvA proteins using the property of RuvA to stimulate RuvB in a classical helicase assay, in which RuvB displaces an oligonucleotide annealed to a ssDNA circular molecule (Figure 5). Both RuvA mutant proteins were capable of RuvB stimulation, but whereas 100 nM RuvA were required for RuvB to unwind 50% of the annealed oligonucleotide, 150 nM of RuvAz87 and 250 nM of RuvAz3 were required. Therefore, RuvAz3 was more deficient than RuvAz87 for the stimulation of the RuvB helicase activity.

**Figure 6:**
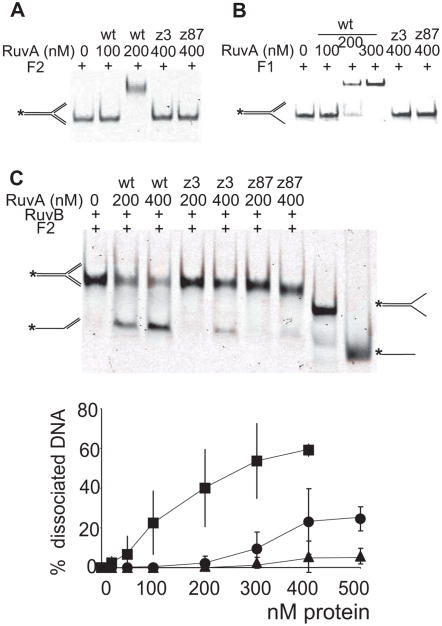
RuvAz3 and RuvAz87 are deficient for fork binding and fork unwinding activity. (A) binding assay: Fluorescence-labeled F2 forks (∼4–5 ng) in which the three DNA arms are double-stranded, were incubated in the presence of 5 mM EDTA with varying amounts of wild-type RuvA or mutant RuvAz3, RuvAz87, as indicated. No binding was detected with mutant RuvA proteins and only the highest concentrations of protein used are shown. (B) as in A but with F1, in which one of the three arms is single-stranded. (C) fork unwinding assay: Reaction mixtures containing ∼5 ng labeled synthetic F2 forks were incubated with 250 nM RuvB and various amounts of wild-type or mutant RuvA, as indicated. The last two lanes contain the controls shown schematically on the right: a fork substrate with two single-stranded daughter arms and the labeled oligonucleotide. Acting on F2, shown schematically on the left, RuvB binds the two arms that mimic the daughter chromosomes and RuvAB unwinds the arm that mimics the template strands. Gels were quantified as in Figure 4. Symbols are as in Figure 4.

To test the binding of the mutants to DNA substrates that mimic replication forks, band shift experiments were performed with an entirely double-stranded fork (F2) or a partially single-stranded fork (F1). Both RuvA mutant proteins were completely defective for fork binding, both in EDTA and in Mg^2+^ buffer (Figure 6A and 6B, data not shown). The single-strand binding protein SSB, which covers ssDNA regions at replication forks *in vivo*, did not stimulate binding of either wild-type or mutant RuvA proteins to forked DNA with an appropriate length of ssDNA regions (not shown). Although it did not act on F1, RuvB unwound F2 in the presence of RuvA (Figure 6C). This reaction was very inefficient in the presence of RuvAz3 and RuvAz87 compared to the wild-type RuvA control (Figure 6C). In conclusion, the two mutated proteins are deficient for fork binding and allow only a weak RuvB action on fork structures. Altogether, these *in vitro* experiments indicate that both mutated proteins are particularly deficient for binding to forked DNA substrates. In addition, RuvAz87 is weakly, and RuvAz3 more strongly affected for RuvB activation.

## Discussion

In this work, we isolated and characterized two *ruvA* mutants that are fully capable of resolving HJs made by homologous recombination after UV irradiation, mitomycin C treatment and Hfr conjugation, while they do not reverse forks at *dnaEts*-blocked forks. In agreement with the mutant strains Rec^+^ phenotype, purified mutant proteins bind HJs nearly as efficiently as wild-type protein. The *in vivo* RFR defect mainly correlates with *in vitro* defects in RuvA octamer formation on HJs and binding to forks. In addition, RuvAz3 is affected for RuvB helicase activation *in vitro*, which could be a major cause of the *ruvAz3* mutant defects *in vivo*, as these defects can be suppressed by over-producing RuvB.

The RuvA polypeptide consists of three distinct domains, I, II and III. The major core domains, I (residues 1–64) and II (residues 65–140), form the central part of the RuvA tetramer and provide a platform for DNA binding. Domain III (residues 156–203), which is linked to domain II by a flexible linker, is involved in RuvB contact and branch migration [10],[15],[33],[34]. Both mutations in *ruvAz87* are in domain II and are very likely to affect primarily DNA binding: N100D lies between two helix-hairpin-helix structures that contact DNA and N79D is within the first of these structures [10],[15] (Figure 2). Mutations in *ruvAz3* affect the three domains (Figure 2). H29R lies within domain I, in a region thought to be involved in RuvA interactions within the tetramer and in DNA binding [33]. K129E is at the end of the second helix-hairpin-helix in domain II, in a region involved in the association of two tetramers to form RuvA octamers [14],[18]. Finally, F140S is the last residue before the flexible linker and may affect the positioning of domain III within the RuvAB complex [35].

Previous studies of RuvA mutants indicated that a combination of three mutations at residues 122, 127 and 130, which disrupt RuvA tetramer-tetramer interface, inactivated recombinational repair *in vivo* [18]. Although the purified proteins do not form octamers on HJs *in vitro*, both *ruvAz3* and *ruvAz87* mutants remain capable of recombinational repair, which suggests that either these mutant proteins form octamers *in vivo*, or octamer formation is not a pre-requisite for homologous recombination. It should be noted that the defect in RuvAz3 and RuvAz87 octamer formation *in vivo* may be responsible for the lack of protection of recombination intermediates from RusA, and may play a role in the RFR defect.

In the complex with a HJ, a RuvA tetramer contacts four double-stranded DNA arms and two RuvB hexamers [34]. In the model that we propose, when RuvAB binds to a replication fork to initiate RFR only three RuvA polypeptides in the tetramer are engaged in DNA contacts, including one with single-stranded DNA, and only one RuvB hexamer is present [8] (Figure 1B). Such a complex might be intrinsically unstable, so that mutant proteins with a decreased DNA affinity would, as the RuvAz mutants described here, retain the ability to bind HJs but lose fork binding. A second RuvA tetramer sandwiching the junction could strengthen interactions with both the fork and the RuvB hexamer. In this case, the defects in octamer formation, fork binding and RuvB activation observed *in vitro* would all contribute to the RFR defect *in vivo*. One of the forces driving the evolution of RuvA, among others such as recombination between diverged sequences or recombination with small DNA fragments, might be to promote RFR. Consequently, RuvA could have acquired a capacity to bind DNA and interact with RuvB exceeding the needs of conjugational recombination and lesion recombinational repair. Our observation that mutations that affect RuvA activity do not necessarily inactivate homologous recombination may explain why, when a collection of 40 *ruvA* mutants was made by alanine replacement of conserved residues, 34 mutants conferred a normal level of UV resistance and 6 only showed a recombinational repair defect [33].

Although RuvAB are among the best-conserved recombination proteins in prokaryotes [9], they do not have close homologues in eukaryotes. The nature of the enzymes that catalyze HJ branch migration and/or resolution in eukaryotes is a subject of debate, possibly because different activities can be involved, depending on the organism and on whether meiotic or mitotic recombination is considered. Rad51 is the functional and structural homologue of RecA in eukaryotes and two mammalian Rad51 orthologues, named Rad51C and XRCC3, were identified as components required for coupled HJ branch migration and resolution in cell extracts [36],[37]. In addition, several purified proteins could catalyze HJ branch migration *in vitro*: the RecQ helicase family members BLM and WRN [38]–[40] and Rad54 [41]. In yeast, the Mus81-Eme1 complex from *Schizosccharomyces pombe* catalyzes the resolution of synthetic HJs *in vitro* and is thought to resolve meiotic recombination intermediates ([42], and references therein). Interestingly, proteins that act on Holliday junctions are most often able to target alternative structures, at least *in vitro*. Mus81, which cleaves nicked HJs, also cleaves fork and D-loop structures (reviewed in [43]). BLM and WRN helicases, which displace HJs, also unwind fork and D-loop structures ([44]; reviewed in [45]). Similarly, the *E. coli* RecG protein promotes HJ branch migration and unwinds D-loops, R-loops and forks [46]–[48]. Recently, the yeast Rad5 protein was shown to promote fork reversal *in vitro* and this reaction may account for the physiological role of Rad5 during post-replicative repair of UV lesions [49]. Fork reversal by Rad5 did not require RPA and did not involve a single-stranded DNA intermediate, which may well be the case with RuvAB. The mammalian BLM and WRN proteins and the bacterial RecG protein were also shown to be able to convert fork structures into HJs *in vitro* [50]–[53]. However, when and where exactly these reactions take place *in vivo* remains to be determined.

It is tempting to speculate that RFR in *E. coli* replication mutants involves interactions of RuvA or RuvB with replication fork-associated proteins. Indeed, several proteins that act at replication forks were shown to interact with fork-associated proteins such as SSB [54] or the polymerase clamp (reviewed in [55]). In addition, RuvB is closely related to clamp-loader subunits, *i.e.* the DNA Pol III δ′ subunit and the replication factor C in eukaryotes [11],[56], and also homologous to RarA (Mgs1 in yeast), a universally conserved protein associated with replication forks [29],[57],[58].

In conclusion, this work shows that it is possible to genetically separate the two functions of the RuvAB complex, RFR and branch migration/resolution of homologous recombination intermediates. Mutations that weaken the function of RuvA inactivate only RFR, possibly because this reaction is more demanding than HJ branch migration.

## Material and Methods

### Strains and Plasmids

Strains were constructed by classical P1 transduction [59] and are described in Table S1. Details of constructions are described in supporting information material. Sequencing of *ruvA* genes in plasmids and chromosome was performed using “Genetic Analyzer” 3100 (Applied Biosystem) automatic sequencer. Oligonucleotides used for sequencing are shown in the supplementary material.

Plasmid constructions are described in supplementary material. For the construction of the mutagenic pGB-ruvAm pool, a protocol derived from Fromant et al, was used [60]. *ruvA* was amplified using a mutagenic PCR reaction containing 10 mM dGTP, 10 mM dCTP, 10 mM dTTP, 2 mM dATP, 5 mM MnCl_2_ and ExTaq (Takara) polymerase. A first denaturation step at 94°C for 10 min was followed by 25 cycles of denaturation at 94°C for 30 s, annealing at 55°C for 30 s, elongation 72°C for 3 min 30 s. The PCR product was purified using Qiagen PCR purification kit and cloned in pGB2 as described. Separations of mutations in pGB-ruvAz60 were done as described in supplementary material.

### Protein Purification

Wild-type RuvA and RuvB proteins were purified as described in previous studies [18]. Some steps were modified or added for the purification of the mutant RuvA proteins, as described in supplementary material. The concentration of RuvB was determined by absorbance at 280-nm wavelength using an extinction coefficient of OD_280,native_ = 16,900 M^−1^ cm^−1^ [61]. All of the other protein concentrations were determined by the Bradford method using the protein assay reagent from Bio-Rad with BSA as a standard.

### DNA Substrates

5′ IRD700-labelled and unlabelled oligonucleotides were purchased from MWG. Oligonucleotide sequences and oligonucleotide assembly are shown in Table S2. Annealing reactions and substrate purification were performed as described [62], using 2 µg of unlabelled and 1 µg of labeled oligonucleotides in each reaction. The helicase substrate was obtained by annealing 10 pmol of IRD-700-labelled 52-mer IT.300 to 10 pmol φx174 virion ssDNA (NEB) in 10 mM Tris-HCl pH 7.5, 10 mM MgCl_2_, 50 mM NaCl [63]. The mixture was denatured at 100°C for 3 min, incubated at 68°C for 30 min and slowly cooled down at room temperature. Purification was performed on a 5–20% sucrose gradient and fractions collected after centrifugation at 4°C, 45000 rpm for 3 h. Substrates were visualized and quantified using the Li-cor Biosciences ODYSSEY infrared imaging system.

### Electrophoretic Mobility Shift Assay

Binding reactions in conditions without magnesium were performed as described [18] and analysed by PAGE in 0.5× Tris-borate EDTA buffer. For binding assays in the presence of magnesium, 3 mM MgCl_2_ was added to the reaction buffer, which did not contain EDTA. Electrophoresis was performed in 0.5× Tris-borate buffer supplemented with 200 µM MgCl_2_ and using buffer recirculation. Reaction products were analyzed using the Li-cor Biosciences ODYSSEY infrared imaging system.

### Branch Migration Assay

Branch migration reactions (20 µl final volume) were performed in 20 mM TrisHCl pH 7.5, 2 mM ATP, 2 mM DTT, 100 µg/ml BSA, 1.5 mM MgCl_2_. The reactions contained ∼5 ng labeled synthetic junction with 250 nM RuvB and various concentrations of RuvA protein. Proteins were diluted in 20 mM Tris HCl pH 7.5, 1 mM EDTA, 0.5 mM DTT, 100 µg/ml BSA, 150 mM NaCl, 10% glycerol. Branch migration reactions were performed as described [18] and visualized as described above.

### DNA Helicase Assay

DNA helicase reactions were performed under the same conditions as branch migration assays described above. Reaction products were analyzed by electrophoresis in 1.2% agarose gel in 1× TAE buffer and visualized as described above.

### UV and Mitomycin C Resistance Tests

UV irradiation was performed as described [8]. For mitomycin C treatment, cells were grown at 37° in LB to an OD_600_ = 0.5 C, mitomycin C was added to the culture at a final concentration of 2 µg/ml and incubation continued at 37°C for 90 minutes. An untreated culture was used as control. Appropriate dilutions were plated on LB plates and incubated over-night at 37°C. Ratios of cfu of mitomycin C treated over cfu of untreated cells were calculated.

### Conjugation

Conjugations were performed as described using JJC145 as Hfr donor [29], donor and recipient cells were mixed for 25 min . Selective medium was M9 minimal medium supplemented with leucine, proline, threonine and arginine (2% final concentration each) and 10 µg/ml Cm.

### Measure of Linear DNA by PFGE

Quantification of pulsed field gels was performed using in vivo ^3^H-thymidine labeled chromosomes as previously described [20].

## Supporting Information

Text S1 Supporting material.
(0.03 MB DOC)

Table S1 Strains.
(0.04 MB DOC)

Table S2 Oligonucleotides.
(0.02 MB DOC)

## References

[1] Lopes M, Foiani M, Sogo JM (2006). Multiple mechanisms control chromosome integrity after replication fork uncoupling and restart at irreparable UV lesions.. Mol Cell.

[2] Kuzminov A (1999). Recombinational repair of DNA damage in Escherichia coli and bacteriophage lambda.. Microbiol Mol Biol Rev.

[3] Michel B, Grompone G, Flores MJ, Bidnenko V (2004). Multiple pathways process stalled replication forks.. Proc Natl Acad Sci U S A.

[4] Michel B, Boubakri H, Baharoglu Z, Lemasson M, Lestini R (2007). Recombination proteins and rescue of arrested replication forks.. DNA Repair (Amst).

[5] Heller RC, Marians KJ (2006). Replisome assembly and the direct restart of stalled replication forks.. Nat Rev Mol Cell Biol.

[6] West SC (1997). Processing of recombination intermediates by the RuvABC proteins.. Ann Rev Genet.

[7] Yamada K, Ariyoshi M, Morikawa K (2004). Three-dimensional structural views of branch migration and resolution in DNA homologous recombination.. Curr Opin Struct Biol.

[8] Baharoglu Z, Petranovic M, Flores MJ, Michel B (2006). RuvAB is essential for replication forks reversal in certain replication mutants.. Embo J.

[9] Rocha EP, Cornet E, Michel B (2005). Comparative and evolutionary analysis of the bacterial homologous recombination systems.. PLoS Genet.

[10] Rafferty JB, Ingleston SM, Hargreaves D, Artymiuk PJ, Sharples GJ (1998). Structural similarities between Escherichia coli RuvA protein and other DNA-binding proteins and a mutational analysis of its binding to the Holliday junction.. J Mol Biol.

[11] Iwasaki H, Han YW, Okamoto T, Ohnishi T, Yoshikawa M (2000). Mutational analysis of the functional motifs of RuvB, an AAA+ class helicase and motor protein for holliday junction branch migration.. Mol Microbiol.

[12] Chamberlain D, Keeley A, Aslam M, ArenasLicea J, Brown T (1998). A synthetic Holliday junction is sandwiched between two tetrameric Mycobacterium leprae RuvA structures in solution: New insights from neutron scattering contrast variation and modelling.. J Mol Biol.

[13] Hargreaves D, Rice DW, Sedelnikova SE, Artymiuk PJ, Lloyd RG (1998). Crystal structure of E-coli RuvA with bound DNA Holliday junction at 6 angstrom resolution.. Nature Struct Biol.

[14] Roe SM, Barlow T, Brown T, Oram M, Keeley A (1998). Crystal structure of an octameric RuvA-Holliday junction complex.. Mol Cell.

[15] Ariyoshi M, Nishino T, Iwasaki H, Shinagawa H, Morikawa K (2000). Crystal structure of the Holliday junction DNA in complex with a single RuvA tetramer.. Proc Nat Acad Sci Usa.

[16] Shinagawa H, Makino K, Amemura M, Kimura S, Iwasaki H (1988). Structure and regulation of the Escherichia coli ruv operon involved in DNA repair and recombination.. J Bacteriol.

[17] Hiom K, Tsaneva IR, West SC (1996). The directionality of RuvAB-mediated branch migration: in vitro studies with three-armed junctions.. Genes Cells.

[18] Privezentzev CV, Keeley A, Sigala B, Tsaneva IR (2005). The role of RuvA octamerization for RuvAB function in vitro and in vivo.. J Biol Chem.

[19] McGlynn P, Lloyd RG (2001). Action of RuvAB at replication fork structures.. J Biol Chem.

[20] Seigneur M, Bidnenko V, Ehrlich SD, Michel B (1998). RuvAB acts at arrested replication forks.. Cell.

[21] Donaldson JR, Courcelle CT, Courcelle J (2006). RuvABC is required to resolve holliday junctions that accumulate following replication on damaged templates in Escherichia coli.. J Biol Chem.

[22] Flores MJ, Sanchez N, Michel B (2005). A fork-clearing role for UvrD.. Mol Microbiol.

[23] Grompone G, Seigneur M, Ehrlich SD, Michel B (2002). Replication fork reversal in DNA polymerase III mutants of Escherichia coli: a role for the beta clamp.. Mol Microbiol.

[24] Shurvinton CE, Lloyd RG, Benson FE, Attfield PV (1984). Genetic analysis and molecular cloning of the Escherichia coli ruv gene.. Mol Gen Genet.

[25] Lloyd RG (1991). Conjugational recombination in resolvase-deficient ruvC mutants of Escherichia coli K-12 depends on recG.. J Bacteriol.

[26] Whitby MC, Vincent SD, Lloyd RG (1994). Branch migration of Holliday junctions: Identification of RecG protein as a junction specific: DNA helicase.. Embo J.

[27] Mandal TN, Mahdi AA, Sharples GJ, Lloyd RG (1993). Resolution of Holliday Intermediates in Recombination and DNA Repair - Indirect Suppression of ruvA, ruvB, and ruvC Mutations.. J Bacterio.

[28] Chan SN, Harris L, Bolt EL, Whitby MC, Lloyd RG (1997). Sequence specificity and biochemical characterization of the RusA Holliday junction resolvase of Escherichia coli.. J Biol Chem.

[29] Lestini R, Michel B (2007). UvrD controls the access of recombination proteins to blocked replication forks.. Embo J.

[30] Tsaneva IR, Illing G, Lloyd RG, West SC (1992). Purification and Properties of the RuvA and RuvB Proteins of Escherichia-Coli.. Mol Gen Genet.

[31] Parsons CA, Tsaneva I, Lloyd RG, West SC (1992). Interaction of Escherichia-Coli RuvA and RuvB Proteins with Synthetic Holliday Junctions.. Proc Natl Acad Sci U S A.

[32] Whitby MC, Bolt EL, Chan SN, Lloyd RG (1996). Interactions between RuvA and RuvC at Holliday junctions: Inhibition of junction cleavage and formation of a RuvA-RuvC-DNA complex.. J Mol Biol.

[33] Nishino T, Ariyoshi M, Iwasaki H, Shinagawa H, Morikawa K (1998). Functional analyses of the domain structure in the Holliday junction binding protein RuvA.. Structure.

[34] Yamada K, Miyata T, Tsuchiya D, Oyama T, Fujiwara Y (2002). Crystal structure of the RuvA-RuvB complex: a structural basis for the Holliday junction migrating motor machinery.. Mol Cell.

[35] Nishino T, Iwasaki H, Kataoka M, Ariyoshi M, Fujita T (2000). Modulation of RuvB function by the mobile domain III of the Holliday junction recognition protein RuvA.. J Mol Biol.

[36] Liu Y, Masson JY, Shah R, O'Regan P, West SC (2004). RAD51C is required for Holliday junction processing in mammalian cells.. Science.

[37] Liu Y, Tarsounas M, O'Regan P, West SC (2007). Role of RAD51C and XRCC3 in genetic recombination and DNA repair.. J Biol Chem.

[38] Constantinou A, Tarsounas M, Karow JK, Brosh RM, Bohr VA (2000). Werner's syndrome protein (WRN) migrates Holliday junctions and co-localizes with RPA upon replication arrest.. EMBO Rep.

[39] Karow JK, Constantinou A, Li JL, West SC, Hickson ID (2000). The Bloom's syndrome gene product promotes branch migration of holliday junctions.. Proc Natl Acad Sci U S A.

[40] Bussen W, Raynard S, Busygina V, Singh AK, Sung P (2007). Holliday Junction Processing Activity of the BLM-Topo III{alpha}-BLAP75 Complex.. J Biol Chem.

[41] Bugreev DV, Mazina OM, Mazin AV (2006). Rad54 protein promotes branch migration of Holliday junctions.. Nature.

[42] Osman F, Dixon J, Doe CL, Whitby MC (2003). Generating crossovers by resolution of nicked Holliday junctions: a role for Mus81-Eme1 in meiosis.. Mol Cell.

[43] Osman F, Whitby MC (2007). Exploring the roles of Mus81-Eme1/Mms4 at perturbed replication forks.. DNA Repair (Amst).

[44] Bachrati CZ, Borts RH, Hickson ID (2006). Mobile D-loops are a preferred substrate for the Bloom's syndrome helicase.. Nucleic Acids Res.

[45] Wu L, Hickson ID (2006). DNA helicases required for homologous recombination and repair of damaged replication forks.. Annu Rev Genet.

[46] McGlynn P, Lloyd RG (1999). RecG helicase activity at three- and four-strand DNA structures.. Nucleic Acids Res.

[47] Vincent SD, Mahdi AA, Lloyd RG (1996). The RecG branch migration protein Escherichia coli dissociates R-loops.. J Mol Biol.

[48] McGlynn P, AlDeib AA, Liu J, Marians KJ, Lloyd RG (1997). The DNA replication protein PriA and the recombination protein RecG bind D-loops.. J Mol Biol.

[49] Blastyak A, Pinter L, Unk I, Prakash L, Prakash S (2007). Yeast rad5 protein required for postreplication repair has a DNA helicase activity specific for replication fork regression.. Mol Cell.

[50] McGlynn P, Lloyd RG (2001). Rescue of stalled replication forks by RecG: Simultaneous translocation on the leading and lagging strand templates supports an active DNA unwinding model of fork reversal and Holliday junction formation.. Proc Natl Acad Sci U S A.

[51] Robu ME, Inman RB, Cox MM (2004). Situational repair of replication forks: roles of RecG and RecA proteins.. J Biol Chem.

[52] Ralf C, Hickson ID, Wu L (2006). The Bloom's syndrome helicase can promote the regression of a model replication fork.. J Biol Chem.

[53] Machwe A, Xiao L, Lloyd RG, Bolt E, Orren DK (2007). Replication fork regression in vitro by the Werner syndrome protein (WRN): holliday junction formation, the effect of leading arm structure and a potential role for WRN exonuclease activity.. Nucleic Acids Res.

[54] Lecointe F, Serena C, Velten M, Costes A, McGovern S (2007). Anticipating chromosomal replication fork arrest: SSB targets repair DNA helicases to active forks.. Embo J.

[55] Friedberg EC, Lehmann AR, Fuchs RP (2005). Trading places: how do DNA polymerases switch during translesion DNA synthesis?. Mol Cell.

[56] Guenther B, Onrust R, Sali A, ODonnell M, Kuriyan J (1997). Crystal structure of the delta' subunit of the cramp-loader complex of E-coli DNA polymerase III.. Cell.

[57] Shibata T, Hishida T, Kubota Y, Han YW, Iwasaki H (2005). Functional overlap between RecA and MgsA (RarA) in the rescue of stalled replication forks in Escherichia coli.. Genes Cells.

[58] Hishida T, Ohya T, Kubota Y, Kamada Y, Shinagawa H (2006). Functional and physical interaction of yeast Mgs1 with PCNA: impact on RAD6-dependent DNA damage tolerance.. Mol Cell Biol.

[59] Miller JH (1992). A short course in bacterial genetic.

[60] Fromant M, Blanquet S, Plateau P (1995). Direct random mutagenesis of gene-sized DNA fragments using polymerase chain reaction.. Anal Biochem.

[61] Hishida T, Iwasaki H, Yagi T, Shinagawa H (1999). Role of Walker motif A of RuvB protein in promoting branch migration of Holliday junctions - Walker motif A mutations affect ATP binding, ATP hydrolyzing, and DNA binding activities of RuvB.. J Biol Chem.

[62] Oram M, Keeley A, Tsaneva I (1998). Holliday junction resolvase in Schizosaccharomyces pombe has identical endonuclease activity to the CCE1 homologue YDC2.. Nucleic Acids Res.

[63] Tsaneva IR, Muller B, West SC (1993). RuvA and RuvB Proteins of Escherichia-Coli Exhibit DNA Helicase Activity Invitro.. Proc Natl Acad Sci U S A.

