# Supplementary material for: ruvA Mutants That Resolve Holliday Junctions but Do Not Reverse Replication Forks
Source: PLoS Genet. 2008 Mar 7;4(3):e1000012. doi: 10.1371/journal.pgen.1000012 (PMC2265524; doi:10.1371/journal.pgen.1000012)
Supplement: Text S1 — Supporting material. (0.03 MB DOC) [file pgen.1000012.s001.doc]

***ruvA* mutants that resolve Holliday junctions but do not reverse replication forks.**

Zeynep Baharoglu^1,2,3^, Alison Sylvia Bradley^4^, Marie Le Masson^1,2,3^, Irina Tsaneva^4^

and Bénédicte Michel*^1,2,3^

**Supporting Material**

Strain construction

For the insertion of the *ruvAz60*-Tet allele into the chromosome, pGB-*ruvAz60* was treated with *Ssp*I and pACYC184 with *Bsr*BI. Digestion reactions were purified using Qiagen PCR purification kit. Ligated DNA was transformed in DH5α and selection was on LB plates containing 7,5 µl/ml tetracycline, to obtain pGB2TC-*ruvAz60*, in which the Tet^R^ gene is inserted ahead of *ruvA*, in the opposite direction. A fragment carrying the Tet^R^ and the *ruvA* genes was amplified by PCR using this plasmid and the following oligonucleotide primers, **GGCAGAAATCAGACGGTCT***GCTTCAATCATCCTTTACC* and **AGTTCATCGAGACACCTCGC***AATCCGTTAGCGAGGTGCC.* The sequences in bold correspond to the chromosome and those in italic amplify the plasmid. The PCR product was purified using the Qiagen PCR purification kit and the insertion in the chromosome was performed by electroporation as described [1].

Plasmid constructions.

pGB-RuvA^+^ : wild-type *ruvA* with its promoter was PCR amplified under standard PCR conditions using pGB-RuvAB [2] as a template and oligonucleotide primers GACGGTAAGCTTCAATCATCC (*Hind*III) and GAATTCCCGGGGATCCTTCG (*Bam*HI) (Tm=55°C), and cloned in pGB2 *Hind*III/*Bam*HI.

pGB-*ruvAz3*-RuvB^+^ was obtained by 3 sequential PCR-based site-directed mutagenesis steps introducing the 3 mutations in plasmid pGB-RuvAB. Mutagenic oligonucleotide pairs were : F1m1 GGTCATCGGCATACGCACTTCATAGCCTACGCCGCCC/ F1m1c GGGCGGCGTAGGCTATGAAGTGCGTATGCCGATGACC; F3m1/F3m1c (mentioned above); F3m2 CCAGGTCGGCGGCTGGCGTAGAGAGATCGCCATGCAAACC / F3m2c GGTTTGCATGGCGATCTCTCTACGCCAGCCGCCGACCTGG.

pGB-*ruvAz87*-ruvB+ was obtained by cloning ruvAz87 in pGB-RuvAB using *Eco*RI+*Bsm*BI restriction enzymes.

pET-r*uvAz3* was obtained by site-directed mutagenesis on template pET21b+-RuvA^+^ as described for pGB-*ruvAz3*-RuvB^+^. pET-*ruvAz87* was obtained by 2 site directed mutagenesis on template pET21b+-RuvA^+^ using the oligonucleotide pairs mut87m1 CAAAGAGTTGATCAAAACCGACGGCGTCGGCCCGAAGTTGGCGC/ mut87m1c GCGCCAACTTCGGGCCGACGCCGTCGGTTTTGATCAACTCTTTG; mut87m2 GTCAGCGCAGCAGTTCGTTGATGCCGTTGAGCGTGAAGAAGTGG /mut87m2c CCACTTCTTCACGCTCAACGGCATCAACGAACTGCTGCGCTGAC.

Sequencing. OBm/OpGB1 and ruvAD/ruvAF oligonulceotides were used for *ruvA* gene sequencing on plasmids and chromosome respectively.

OBam GAATTCCCGGGGATCCTTCG / OpGB1 CGAAGTAATCGCAACATCCGC

ruvAD GATAGGCAGACTCAGAGG / ruvAF CATAACGCGGCGCGTAGG

Separation of mutations of pGB*-ruvAz60*

The restriction enzymes used for each fragment were *Eco*RI+*Pfl*MI for F1, *Pfl*MI+*Bsp*EI for F2, *Bsp*EI+*Sex*AI for F3, *Sex*AI+*Hind*III for F4. A wild-type fragment was cloned in pGB-*ruvAz60* to obtain pF1+, pF2+, pF3+, pF4+ and a mutant fragment was cloned in pGB-RuvA^+^ to obtain pF1m, pF2m, pF3m, pF4m. pF1mF3m was obtained by cloning F3m in pF1m. pF1m1-F3m, pF1m1-F3m1, pF1m1-F3m2 and pF1m2-F3m were obtained by site-directed mutagenesis using Stratagene QuikChange Site directed mutagenesis kit and the oligonucleotides shown below.

| Template plasmid | Obtained plasmid | Oligonucleotides 5’-3’ |
| --- | --- | --- |
| pF1m-F3m | pF1m1-F3m | F1wt2 CGCTTCCTGACCCGCTTCAGGGAGTTCATAAAAAC  F1wt2c GTTTTTATGAACTCCCTGAAGCGGGTCAGGAAGCG |
| pF1m-F3m | pF1m2-F3m | F1wt1 CAGGTCATCGGCATATGCACTTCATAGCCTACGC  F1wt1c GCGTAGGCTATGAAGTGCATATGCCGATGACCTG |
| pF1m | pF1m-F3m1 | F3m1 CCATGCAAACCTTTAAATCGGTCCTCCATTTCAACAATCAAGCG  F3m1c CGCTTGATTGTTGAAATGGAGGACCGATTTAAAGGTTTGCATGG |
| pF1m-3m1 | pF1m1-3m1 | F1wt2 CGCTTCCTGACCCGCTTCAGGGAGTTCATAAAAAC  F1wt2c GTTTTTATGAACTCCCTGAAGCGGGTCAGGAAGCG |
| pF1m1-3m | pF1m1-3m2 | F3wt1 GCAAACCTTTAAATCGGTCTTTCATTTCAACAATCAAGCGTTCG  F3wt1c CGAACGCTTGATTGTTGAAATGAAAGACCGATTTAAAGGTTTGC |

Purification of mutant RuvA proteins.

Mutant RuvA proteins were over expressed from the pET21*ruvA* constructs in *E. coli* BL21-Gold (DE3). The culture was grown at 37 °C in LB medium supplemented with ampicillin (100 µg/ml) and 1% glucose to OD_600_ nm = 0.8 and induced with 1 mM IPTG for 4 h at 37 °C. Bacteria were collected by centrifugation, washed in lysis buffer (100 mM Tris-HCl, pH 8.0, 2 mM EDTA, 5% glycerol), resuspended in the same buffer, and frozen. The thawed cell suspension was treated with 1 mM DTT and 1 mg/ml lysozyme for 30 min on ice. The lysate was made 1M in NaCl and 0.1% Triton and incubated on ice for 10 min. The resulting suspension of lysed cells was supplemented with sodium deoxycholate 0.4% and cleared by centrifugation in a Beckman Ti70 rotor at 42,000 rpm for 1 h at 4°C. The supernatant containing crude extract was dialyzed against TEGD A Buffer (20 mM Tris-HCl, pH8.5, 1 mM EDTA, 10% glycerol, 0.5 mM DTT). For purification of RuvAz87 protein, the dialyzed crude extract was applied on DEAE column (BioRad) equilibrated with TEGD buffer. The column was eluted with a linear gradient of 0-500 mM KCl in the same buffer. Fractions containing RuvA were dialyzed against TEGD supplemented with 150 mM KCl. The dialyzed fractions were applied to ssDNA cellulose column (Sigma Aldrich) equilibrated with TEGD 150 mM KCl. The column was eluted with a linear gradient of 150-1000 mM KCl in TEGD buffer. The fractions containing RuvA were pooled and dialyzed against P100 buffer (10 mM KPi pH 6.8, 10% glycerol, 5 mM β-mercaptoethanol, 0.1 mM PMSF, 100 mM KCl). The dialyzed protein solution was loaded onto HiTrap Heparin HP column (GE Healthcare) equilibrated with the same buffer, and the column was developed with a linear gradient from 100 to 1000 mM KCl in P100 buffer. The mutant RuvAz3 protein was expressed and fractionated by the procedures described above up to the second purification step. Fractions eluted from the DEAE column were pooled and dialyzed against phosphate buffer (10 mM KPi, 0.5 mM DTT, 10% glycerol) with 150 mM KCl. Instead of ssDNA cellulose column, the dialyzed fractions were applied to a Hydroxyapatite Bio-Gel HTP (Bio-Rad) column pre-equilibrated in the same buffer. The proteins were eluted with a 10–600 mM linear gradient of potassium phosphate. The fractions containing RuvA were pooled and dialyzed against P100 buffer and a third purification step on HiTrap Heparin HP column (GE Healthcare) was performed as described above. To increase purity, the fractions containing RuvAz3 were dialyzed against TEGD buffer and loaded onto HiTrap Q HP column, and the protein was eluted with a linear gradient from 100 to 1000 mM KCl.

References

1. Datsenko KA, Wanner BL (2000) One-step inactivation of chromosomal genes in Escherichia coli K-12 using PCR products. Proc Natl Acad Sci U S A 97: 6640-6645.

2. Seigneur M, Bidnenko V, Ehrlich SD, Michel B (1998) RuvAB acts at arrested replication forks. Cell 95: 419-430.
