# Supplementary material for: ruvA Mutants That Resolve Holliday Junctions but Do Not Reverse Replication Forks
Source: PLoS Genet. 2008 Mar 7;4(3):e1000012. doi: 10.1371/journal.pgen.1000012 (PMC2265524; doi:10.1371/journal.pgen.1000012)
Supplement: Table S1 — Strains. (0.04 MB DOC) [file pgen.1000012.s002.doc]

***ruvA* mutants that resolve Holliday junctions but do not reverse replication forks.**

Zeynep Baharoglu^1,2,3^, Alison Sylvia Bradley^4^, Marie Le Masson^1,2,3^, Irina Tsaneva^4^

and Bénédicte Michel*^1,2,3^

**Supporting Material**

Table S1 Strains

| Strain | Relevant Genotype | Construction or reference |
| --- | --- | --- |
| JJC40 | *wild-type* | As AB1157 but *hsdR* Thr^+^ Pro^+^ |
| JJC145 | Hfr PK19-PO66 Δ*-gpt-lac)supE44 srlC::Tn10 thi1* | CGSC6813 Genetic Stock Center |
| JJC944 | Δ*recG263*::kan | N3793 = CF3324 in [2] |
| JJC1193 | *recR252*::Tn*10*kan | AM207 in [3] |
| JJC1263 | *sfiA11 recB270ts recC271ts* | *sfiA11* derivative of SK129 in [4] |
| JJC1321 | *dnaE486ts zae3095*::Tn*10*kan | [5] |
| JJC1371 | pDK46 [oriR101] [repA101(ts)] arap-gam-bet-exo Ap100 | BW25113 [pHD46] in [6] |
| JJC1377 | Δ*recBC*::Ap [pGB-RecBCD^+^] | LN2666 in [7] transformed with pGB-RecBCD^+^ |
| JJC2211 | *sfiA11 recB270ts recC271ts dnaE486ts zae3095*::Tn*10*kan | [8] |
| JJC2721 | *sfiA11 recB270ts recC271ts ruvA60*::Tn*10 rus-1* | [8] |
| JJC2750 | *sfiA11 dnaE486ts zae502*::Tn*10 recF400*::Tn*5* | [9] |
| JJC2761 | *sfiA11* Δ*ruvABC::Cm rus-1* | [10] |
| JJC2971 | Δ*ruvA100::*cat | HRS2300 in [11] |
| JJC3105 | *sfiA11 dnaE486ts zae502*::Tn*10 recF400*::Tn*5* Δ*ruvA100::cm* | JJC2750*P1 JJC2971 |
| JJC3110 | *sfiA11 dnaE486ts zae502*::Tn*10 recF400*::Tn*5* Δ*ruvA100::cm* Δ*recBC*::Ap | JJC3105*P1 JJC1377 |
| JJC3207 | Δ*ruvA100::*cat Δ*recG263*::kan | JJC2971* P1 JJC944 |
| JJC3375 | Δ*ruvA100::*cat recR::kan | JJC2971*P1 JJC1193 |
| JJC3412 | *ruvAz60*-Tet | Integration of *ruvAz60*-Tet in JJC1371 chromosome (described below) |
| JJC3495 | *sfiA11 recB270ts recC271ts ruvAz60*-Tet | JJC1263*P1 JJC3412 |
| JJC3723 | *sfiA11 recB270ts recC271ts dnaE486ts zae3095*::Tn*10*kan Δ*ruvA100::*cat | JJC2211*P1 JJC2971 |
| JJC3939 JJC4015 | *sfiA11 recB270ts recC271ts ruvAz60*-Tet *dnaE486ts zae3095*::Tn*10*kan | JJC3495*P1 JJC1321 |
| JJC4177 | *sfiA11 recB270ts recC271ts ruvA60*::Tn*10 rus-1* Δ*ruvA100::*cat | JJC2721*P1 JJC2971 |
| JJC4196 | *sfiA11 recB270ts recC271ts ruvA60*::Tn*10 rus-1* Δ*ruvA100::cm dnaE486ts zae3095*::Tn*10*kan | JJC4177*P1 JJC1321 |

References

1. Nishino T, Ariyoshi M, Iwasaki H, Shinagawa H, Morikawa K (1998) Functional analyses of the domain structure in the Holliday junction binding protein RuvA. Structure 6: 11-21.

2. Mandal TN, Mahdi AA, Sharples GJ, Lloyd RG (1993) Resolution of Holliday Intermediates in Recombination and DNA Repair - Indirect Suppression of ruvA, ruvB, and ruvC Mutations. JBacteriol 175: 4325-4334.

3. Mahdi AA, Lloyd RG (1989) Identification of the recR locus of Escherichia coli K-12 and analysis of its role in recombination and DNA repair. Mol Gen Genet 216: 503-510.

4. Kushner SR (1974) In vivo studies of temperature-sensitive recB and recC mutants. J Bacteriol 120: 1213-1218.

5. Grompone G, Seigneur M, Ehrlich SD, Michel B (2002) Replication fork reversal in DNA polymerase III mutants of Escherichia coli: a role for the beta clamp. Mol Microbiol 44: 1331-1339.

6. Datsenko KA, Wanner BL (2000) One-step inactivation of chromosomal genes in Escherichia coli K-12 using PCR products. Proc Natl Acad Sci U S A 97: 6640-6645.

7. Corre J, Cornet F, Patte J, Louarn JM (1997) Unraveling a region-specific hyper-recombination phenomenon: Genetic control and modalities of terminal recombination in Escherichia coli. Genetics 147: 979-989.

8. Lestini R, Michel B (2007) UvrD controls the access of recombination proteins to blocked replication forks. Embo J 26: 3804-3814.

9. Flores MJ, Sanchez N, Michel B (2005) A fork-clearing role for UvrD. Mol Microbiol 57: 1664-1675.

10. Baharoglu Z, Petranovic M, Flores MJ, Michel B (2006) RuvAB is essential for replication forks reversal in certain replication mutants. Embo J 25: 596-604.

11. Hishida T, Iwasaki H, Ishioka K, Shinagawa H (1996) Molecular analysis of the Pseudomonas aeruginosa genes, ruvA, ruvB and ruvC, involved in processing of homologous recombination intermediates. Gene 182: 63-70.
