# Supplementary material for: ruvA Mutants That Resolve Holliday Junctions but Do Not Reverse Replication Forks
Source: PLoS Genet. 2008 Mar 7;4(3):e1000012. doi: 10.1371/journal.pgen.1000012 (PMC2265524; doi:10.1371/journal.pgen.1000012)
Supplement: Table S2 — Oligonucleotides. (0.02 MB DOC) [file pgen.1000012.s003.doc]

***ruvA* mutants that resolve Holliday junctions but do not reverse replication forks.**

Zeynep Baharoglu^1,2,3^, Alison Sylvia Bradley^4^, Marie Le Masson^1,2,3^, Irina Tsaneva^4^

and Bénédicte Michel*^1,2,3^

**Supporting Material**

Table S2 Oligonucleotides used for the construction of *in vitro* DNA substrates.

|  | 5’ 🡪 3’ |
| --- | --- |
| IT.01  61-mer | IRD 700 - GACGCTGCCGAATTCTACCAGTGCCTTGCTAGGACATCTTTGCCCACCTGCAGGTTCACCC |
| IT.04  62-mer | ATGGATAGTCGGATCCTCTAGACAGCTCCATGTAGCAAGGCACTGGTAGAATTCGGCAGCGT |
| IT.06A  31-mer | TGGGTGAACCTGCAGGTGGGCAAAGATGTCC |
| IT.06B  31-mer | CATGGAGCTGTCTAGAGGATCCGACTATCGA |
| IT.07  62-mer | TGGGTGAACCTGCAGGTGGGCAAAGATGTCCTAGCAATGTAATCGTCAAGCTTTATGCCGTT |
| IT.08  63-mer | CAACGGCATAAAGCTTGACGATTACATTGCTAGGACATGCTGTCTAGAGGATCCGACTATCGA |
| IT.09  62-mer | ATCGATAGTCGGATCCTCTAGACAGCATGTCCTAGCAAGGCACTGGTAGAATTCGGCAGCGT |
| IT.300 | IRD 700 - ATGGAGCTGCGCAAGGATAGGTCGAA |

Oligonucleotides were assembled to get different substrates as follows :

**X12:** IT.01+IT.07+IT.08+IT.09; **F1:** IT.01+IT.04+IT.06A; **F2:** IT.01+IT.04+IT.06A+IT.06B
